# Temporal differences in the development of organ dysfunction based on two different approaches to induce experimental intra-abdominal hypertension in swine

**DOI:** 10.1186/2110-5820-2-S1-S16

**Published:** 2012-07-05

**Authors:** Michael Moller, Ulrik K Kjerkegaard, Jens Rolighed Larsen, Hanne Birke-Sorensen, Lars B Stolle

**Affiliations:** 1 Institute of Clinical Medicine, Aarhus University, Aarhus University Hospital, Skejby, Brendstrupgaardsvej 100, Aarhus N, 8200, Denmark; 2 Aarhus University, Aarhus C, 8000, Denmark; 3 T-research, Department of Cardiothoracic and Vascular Surgery, Aarhus University Hospital, Skejby, Brendstrupgaardsvej 100, Aarhus N, 8200, Denmark; 4 Department of Anesthesiology-Intensive Care, Aarhus University Hospital, Skejby, Brendstrupgaardsvej 100, Aarhus N, 8200, Denmark; 5 Department of Plastic Surgery, Aarhus University Hospital, Aarhus C, 8000, Denmark

**Keywords:** multiple organ failure, multiple organ dysfunction syndrome, swine, acute kidney injury, acidosis, abdomen.

## Abstract

**Background:**

Intra-abdominal hypertension [IAH] occurs frequently among critically ill patients and is associated with increased mortality and organ failure. Two porcine models of IAH that cause abdominal compartment syndrome [ACS] with organ dysfunction were created. We investigated whether the two methods used to create IAH - CO_2 _pneumoperitoneum or adding volume to the intra-abdominal space - exerted different impacts on the temporal development of organ dysfunction.

**Methods:**

Twenty-four 40-kg female pigs were allocated to four groups: 25 mmHg IAH with CO_2 _pneumoperitoneum (*n ***= **8), **>**20 mmHg IAH caused by addition of volume (*n ***= **8), and two corresponding sham groups (each *n ***= **4). The two sham groups were later pooled into one control group (*n ***= **8). The animals were monitored for 12 h. Repeated serial measurements were taken of group differences over time and analyzed using analysis of variance.

**Results:**

Thirty-eight percent of the animals (*n ***= **3) in each intervention group died near the end of the 12-h experiment. Both intervention groups experienced kidney impairment: increased creatinine concentration (*P ***<**0.0001), anuria (*P ***= **0.0005), hyperkalemia (*P ***<**0.0001), decreased abdominal perfusion pressure, and decreased dynamic lung compliance. CO_2 _pneumoperitoneum animals developed hypercapnia (*P ***<**0.0001) and acidosis (*P ***<**0.0001).

**Conclusions:**

Both methods caused ACS and organ dysfunction within 12 h. Hypercapnia and acidosis developed in the CO_2 _pneumoperitoneum group.

## Background

The multiple organ dysfunction syndrome [MODS] is a major cause of death among patients with infection/sepsis and traumatic or surgical injury [1]. Intra-abdominal hypertension [IAH] occurs frequently among critically ill patients and is both a predictor of MODS [2] and associated with mortality and organ failure [3,4]. An increase in intra-abdominal pressure [IAP] > 20 mmHg and associated new organ damage are referred to as the abdominal compartment syndrome [ACS] [5]. Prior studies have shown that risk factors for the development of IAH and ACS include acute respiratory failure, abdominal surgery, sepsis, massive fluid resuscitation, and major trauma [6].

It is difficult to develop a single experimental animal model of MODS based on IAH, and unfortunately, the ideal model of ACS is not yet developed [7]. CO_2 _pneumoperitoneum generated by laparoscopic insufflation was used to generate IAH in experimental models [8-12]. CO_2 _pneumoperitoneum is known to impact the immune response in at least two ways: as a direct consequence of the peritoneal acidosis [13] and by causing systemic acidosis [14], which also influences the immune response [15]. Fluid-filled bags placed intra-abdominally were used to simulate IAH [16]. Both methods generate IAH and cause ACS, and might therefore produce MODS. The question remains whether there are differences in the effects that the models have on organ dysfunction.

The main aim of this study was to investigate differences in creating IAH by CO_2 _pneumoperitoneum or by adding volume to the intra-abdominal space using a fluid bag model. With IAH generated by laparoscopic CO_2 _insufflations in one model and volume added to the intra-abdominal cavity in another model, we could evaluate and compare the impacts on the kidneys, cardiovascular system, metabolism, and mortality.

## Materials and methods

The investigation conformed to the Danish law for animal research (Act no. 1306 of 23/11/2007, Danish Ministry of Justice) and the guidelines from *Guide for the Care and Use of Laboratory Animals*, published by the U.S. National Institutes of Health (NIH Publication No. 85-23, revised 1996).

### Experimental setup

The study was an experimental animal study performed at a university research laboratory. Twenty-four pigs were allocated to four groups: IAH with CO_2 _pneumoperitoneum [CO2] (*n *= 8), CO_2 _pneumoperitoneum sham (*n *= 4), IAH with intra-abdominal volume addition [VOL] (*n *= 8), and intra-abdominal volume addition sham (*n *= 4). After all experiments were performed, we evaluated the data from the CO2 sham group and the VOL sham group at *T *= 0 h and *T *= 10 h for the following parameters: weight, diuresis, pH, partial pressure of CO_2 _[pCO_2_], base excess, K^+^, lactate, heart rate, mean arterial pressure [MAP], mean pulmonary artery pressure [MPAP], central venous pressure [CVP], and creatinine. The groups were similar for all measured values except lactate *T *= 0 h (mean value for the three groups: control = 0.963, CO2 = 0.863, VOL = 1.125) (*P *= 0.03). Data from the CO2 sham group and the VOL sham group were therefore pooled into one control group (*n *= 8). After baseline measurements (*T *= 0 h), the pigs were randomized to one of the four study groups, and the IAH induction procedure or the sham operation was performed. Sampling started at baseline (*T *= 0 h) and continued with one sample per hour for 12 h (*T *= 1 h to *T *= 12 h). The following data were collected: (1) physiological parameters: intra-bladder pressure [IBP], heart rate, MAP, MPAP, CVP, abdominal perfusion pressure (APP = MAP - IAP), and tidal volume; (2) arterial blood samples: pH, pCO_2_, base excess, K^+^, and lactate; and (3) venous blood samples: creatinine.

### Anesthesia

The animals were female Danish Landrace pigs that had fasted for 24 h prior to the experiment and had free access to water. Prior to transportation to the institute, they received 0.5 mg/kg midazolam (Janssen Pharmaceutica, Beerse, Belgium) and 4 mg/kg azeperone (Janssen-Cilag GmbH, Neuss, Germany) intramuscularly. General anesthesia was induced with an intramuscular injection of 4.35 mg/kg (S)-ketamine (Pfizer ApS, Ballerup, Denmark) and 0.375 mg/kg midazolam (Hameln Pharmaceuticals GmbH, Hameln, Germany) intravenously, followed by intubation. After intubation, the animals were ventilated via a respirator (Datex-Ohmeda S/5 Avance, GE Healthcare, Brøndby, Denmark). Sedation was maintained with sevoflurane (Abbott Scandinavia AB, Solna, Sweden) inhalation to obtain a minimal alveolar concentration of approximately 1.5. Fentanyl was infused intravenously at a constant rate of 12.5 μg/kg/h. No muscular relaxant was used. Pressure-controlled respirator settings were used. Initial settings were a fraction of inspired oxygen [FiO_2_] of 0.3; positive end-expiratory pressure [PEEP] of 4 cm H_2_0; inspiratory pressure [*P*_insp_] of 12 cm H_2_O, restricting peak airway pressure to a maximum of 16 cm H_2_O; respiratory frequency of 12/min, and inhalation/expiration ration [I/E] of 1:2. Saline was infused at a constant rate of 1.5 ml/kg/h.

A 6Fr catheter was placed in the carotid artery using a cutdown technique for the measurement of arterial blood pressure and arterial blood sampling. An 8Fr catheter was placed in the right external jugular vein for infusions and to introduce a Swan-Ganz catheter (CCOmbo^®^, Edwards Lifesciences LLC, Irvine, CA, USA) connected to a Baxter Vigilance monitor (Edwards Life Sciences LLC, Irvine, CA, USA) in order to measure MPAP and CVP. A 7Fr catheter was placed in the left external jugular vein for venous blood sampling. The urinary bladder was catheterized using a 12G Foley catheter connected to an IBP measuring catheter (UnoMeter Abdo-Pressure, Unomedical, Birkerød, Denmark) and a sample tube to monitor diuresis. A rectal thermometer was inserted to measure core temperature. A pulse oximetry device was attached to the pig's tail to observe arterial blood oxygen saturation.

### Induction of intra-abdominal hypertension

#### Induction of IAH with the CO2 group

Verres Cannula was inserted below the umbilicus and attached to a laparoscopic CO_2 _insufflator (Thermoflator 26432020, Karl Storz, Holte, Denmark or Vision F103, Lemke, Berlin, Germany). The insufflator was set to IAP = 25 mmHg.

#### Induction of IAH with the VOL group

Via a 15-cm incision above the umbilicus, seven 1-l ordinary saline infusion bags were placed in the abdominal cavity. Bags were positioned into the small pelvis until IBP was above 20 mmHg. The abdominal wall was closed in two layers, including the fascia, using running sutures.

#### CO_2 _pneumoperitoneum sham group

These animals underwent a procedure similar to the CO2 group, but Verres Cannula remained unattached to the insufflator.

#### Intra-abdominal volume addition sham group

The animals underwent a procedure similar to the VOL group, but empty saline bags were inserted into the abdominal cavity.

### Anesthesia maintenance and ventilator settings during experiment

IAH decreases lung capacity and requires the adjustment of respirator settings to ensure sufficient ventilation. Our priorities were to maintain arterial oxygen saturation > 90%, pCO_2 _< 5 kPa, MAP > 60 mmHg, and a tidal volume of 400 ml. Initial respirator settings could be increased up to F_i_O_2 _= 0.7; PEEP = 8 cm H_2_O; *P*_insp _= 24 cm H_2_O, restricting peak airway pressure to a maximum of 32 cm H_2_O; respiratory frequency = 24; and I/E = 1:1.5. Immediately following IAH generation, all respirator settings, except F_i_O_2_, were adjusted to maximally allowed settings in order to avoid respiratory complications such as atelectasis. A 500-ml bag of Rheomacrodex^® ^100 mg/ml with saline was infused when arterial pressure was < 60 mmHg. Two bags of Rheomacrodex^® ^(Meda A/S, Alleroed, Denmark) were allowed for each pig.

### Physiologic measurements

A fixed point at bladder level was marked on the animals. Prior to IBP measurements, the bladder pressure-measuring catheter was elevated. This installed the fluid inside the catheter into the bladder, ensuring that the bladder was not empty when IBP was measured. The zero point of the IBP-measuring catheter was aligned with the marked point on the pig, and the catheter was held vertically. End-expiratory bladder pressure was noted. The animals were in supine position.

Arterial blood samples were analyzed on a blood gas analyzer (ABL 700 series, Radiometer, Copenhagen, Denmark). Heart rates and blood pressures were sampled and recorded through a computer connected to the respirator using software (Datex-Ohmeda S5 Collect Program, version 4.0, Datex-Ohmeda Division, Instrumentarium Corp, Helsinki, Finland). Venous blood samples were stored at -20°C, and the creatinine content was measured by trained personnel at Aarhus University Hospital. APP was calculated as MAP - IBP. Dynamic lung compliance [Cdyn] was calculated as tidal volume/(*P*_insp _- PEEP).

### Statistics

Repeated serial measurements were tested for group differences over time by univariate repeated measures using analysis of variance [ANOVA]. MedCalc 9.3.2.0 (MecCalc Software, Mariakerke, Belgium) and Intercooled STATA 9.2 (College Station, TX, USA) were used in the analyses. Furthermore, a Kruskal-Wallis one-way ANOVA on ranks using Dunn's method for multiple comparisons was performed between the three groups at corresponding moments. Sigmastat™ version 3.5 (Systat Software, San Jose, California, USA) was used in the analyses. The similarity between the two sham groups (CO_2 _pneumoperitoneum sham group (*n *= 4) and the intra-abdominal volume addition sham group (*n *= 4)) was tested with the Mann-Whitney rank sum test using SigmaStat™ version 3.11 (Systat Software, San Jose, California, USA) at two points: *T *= 0 h and *T *= 10 h. For all analyses, a *P *value of less than 0.05 was considered significant.

## Results

### Baseline values

No difference at baseline was observed between the three groups: control (*n *= 8), CO2 (*n *= 8), and VOL (*n *= 8) (Tables 1 and 2).

**Table 1:** Physiological parameters.

Parameter	Group	*T *= 0 h (baseline)^a^	*T *= 12 h^a^	*P *value^b^
IBP (mmHg)	Control	5 (2)	7 (1)	< 0.0001
	CO2	5 (2)	26 (1)^c^	
	VOL	5 (1)	21 (4)	
Survival (%)	Control	100	100	
	CO2	100	63	
	VOL	100	63	
pCO_2 _(kPa)	Control	4.6 (0.6)	5.4 (0.9)	< 0.0001
	CO2	4.9 (0.4)	11.5 (3.2)^c^	
	VOL	4.5 (0.5)	7.4 (4.5)	
pH	Control	7.5 (0.1)	7.5 (0.1)	< 0.0001
	CO2	7.5 (0.0)	7.1 (0.1)^c^	
	VOL	7.6 (0.0)	7.3 (0.2)	
Base excess (mmol/l)	Control	7.0 (0.8)	4.4 (1.3)	< 0.0001
	CO2	7.0 (1.2)	-1.4 (4.7)^c^	
	VOL	7.5 (1.3)	-0.1 (4.6)	
Creatinine (mg/dl)	Control	1.4 (0.3)	1.9 (0.5)	< 0.0001
	CO2	1.4 (0.3)	3.8 (0.7)^c^	
	VOL	1.3 (0.3)	4.3 (0.8)^c^	
Diuresis (ml/h)	Control	48 (38)	38 (22)	0.0005
	CO2	43 (24)	1 (2)^c^	
	VOL	49 (27)	4 (4)^c^	
K^+ ^(mmol/l)	Control	4.7 (1.0)	4.6 (0.5)	< 0.0001
	CO2	4.4 (0.5)	7.6 (1.1)^c^	
	VOL	4.1 (0.3)	8.2 (0.4)^c^	
Lactate (mmol/l)	Control	1.0 (0.3)	0.8 (0.4)	0.0007
	CO2	0.9 (0.2)	3.6 (3.1)	
	VOL	1.1 (0.4)	3.1 (3.3)^c^	

^a^Data presented as mean ± standard deviation [SD]. Control, control group; CO2, group with CO_2 _pneumoperitoneum; VOL, group with intra-abdominal volume addition by placement of saline bags. Statistics: group-time interactions were analyzed by repeated measures ANOVA. ^b^Difference between groups over time. Kruskal-Wallis one-way ANOVA on ranks using Dunn's method for multiple comparisons between groups at corresponding moments, ^c^different from control,. For survival, no statistically significant different odds ratios were observed: control vs. VOL = 10.8 (*P *= 0.14), control vs. CO2 = 6.5 (*P *= 0.25), VOL vs. CO2 = 1.8 (*P *= 0.59). IBP, intra-bladder pressure; pCO_2_, partial pressure of CO_2_.

**Table 2:** Hemodynamic and pulmonary parameters.

Parameter	Group	*T *= 0 h (baseline)^a^	*T *= 12 h^a^	*P *value^b^
Heart rate (beats/min)	Control	71 (13)	92 (28)	< 0.0001
	CO2	81 (23)	140 (35)	
	VOL	65 (10)	91 (14)	
CVP (mmHg)	Control	3 (2)	5 (3)	< 0.0001
	CO2	2 (3)	9 (2)	
	VOL	5 (3)	16 (7)^c^	
MPAP (mmHg)	Control	11 (4)	17 (6)	0.0195
	CO2	12 (3)	27 (10)	
	VOL	13 (3)	27 (9)	
MAP (mmHg)	Control	59 (10)	60 (3)	0.0012
	CO2	58 (11)	57 (5)	
	VOL	68 (12)	56 (11)	
APP (mmHg)	Control	53 (9)	55 (4)	< 0.0001
	CO2	54 (11)	31 (6)^c^	
	VOL	64 (12)	39 (3)^c^	
Cdyn (ml/cm H_2_O)	Control	33 (4)	26 (5)	< 0.0001
	CO2	33 (5)	8 (2)^c^	
	VOL	34 (2)	12 (4)^c^	

^a^Data presented as mean ± SD. Control, control group; CO2, group with CO_2 _pneumoperitoneum; VOL, group with intra-abdominal volume addition by placement of saline bags. Statistics: group-time interactions were analyzed by repeated measures ANOVA. ^b^*P *value, difference between groups over time. Kruskal-Wallis one-way ANOVA on ranks using Dunn's method for multiple comparisons between groups at corresponding moments, ^c^different from control, CVP, central venous pressure; MPAP, mean pulmonary artery pressure; MAP, mean arterial pressure; APP, abdominal perfusion pressure; Cdyn, dynamic lung compliance.

### Intra-bladder pressure and survival

IBP in the control group was lower than that in the intervention groups (*P *< 0.0001, repeated measures ANOVA. VOL group IBP differed from control at most other times than *T *= 12 h (*P *< 0.05, Kruskal-Wallis), data not presented). In both intervention groups, IBP was above 20 mmHg and remained stable. CO2 group mean IBP was constantly 1 to 2 mmHg above the 25 mmHg IAP to which the laparoscopic insufflators were adjusted. No animals died in the control group during the experiment. Three animals (38%) died in each intervention group (Table 1).

### Acid-base status

**Figure 1:**
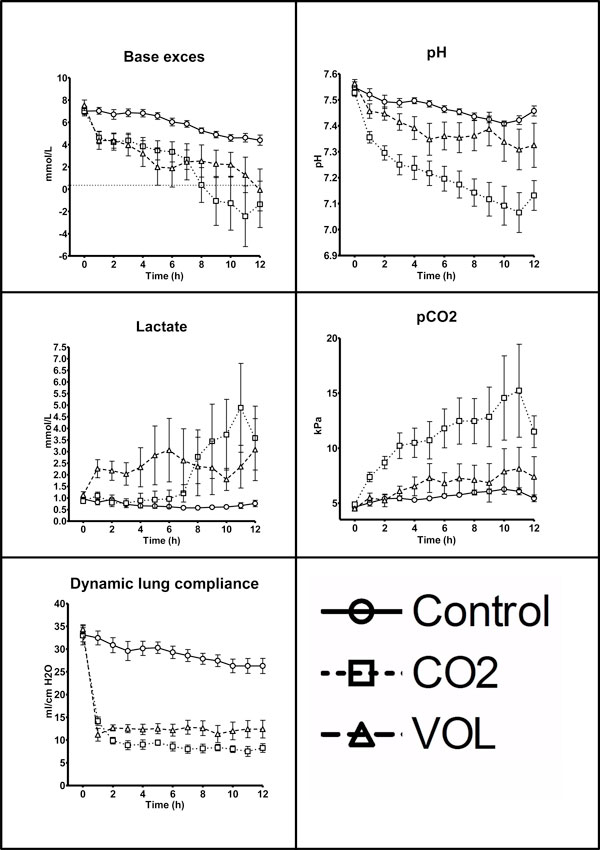
**Metabolic and respiratory parameters**. Data presented as mean ± standard error of the mean [SEM]. Control, control group; CO2, group with CO_2 _pneumoperitoneum; VOL, group with intra-abdominal volume addition by placement of saline bags.

The pigs in the CO2 group showed a higher increase in pCO_2 _and decrease in pH than pigs in the two other groups throughout the experiment. Both intervention groups experienced decreasing base excess; for the CO_2 _group, this was observed throughout the experiment (*P *< 0.0001, repeated measures ANOVA. VOL group base excess differed from control until *T *= 7 h (*P *< 0.05, Kruskal-Wallis), data not presented) (Table 1, Figure 1).

### Organ impact

**Figure 2:**
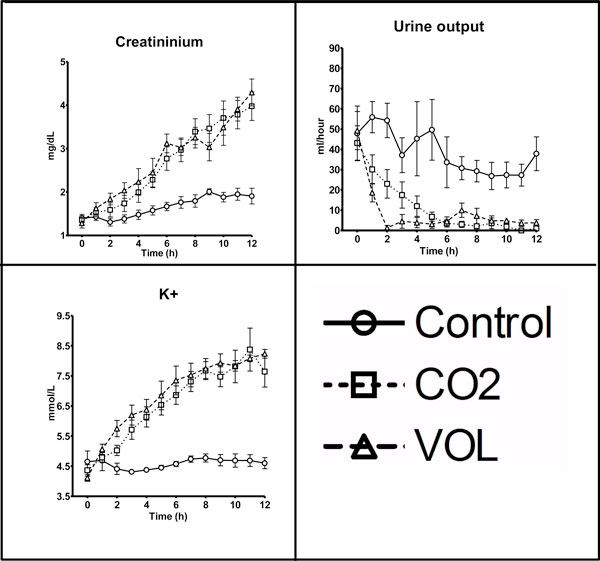
**Renal parameters**. Data presented as mean ± SEM. Control, control group; CO2, group with CO_2 _pneumoperitoneum; VOL, group with intra-abdominal volume addition by placement of saline bags.

Kidney function was affected in a similar manner in both intervention groups, with increasing creatinine concentration and decreasing urine output, ending in anuria (*P *< 0.0001, repeated measures ANOVA) (Table 1). Similarly, increases in K^+ ^concentration (*P *< 0.0001, repeated measures ANOVA) and lactate concentration (*P *= 0.0007, repeated measures ANOVA. CO2 group lactate was different from control at *T *= 10 to 11 h (*P *< 0.05, Kruskal-Wallis), data not presented) were found in both intervention groups (Table 1, Figure 2).

### Circulatory effects

**Figure 3:**
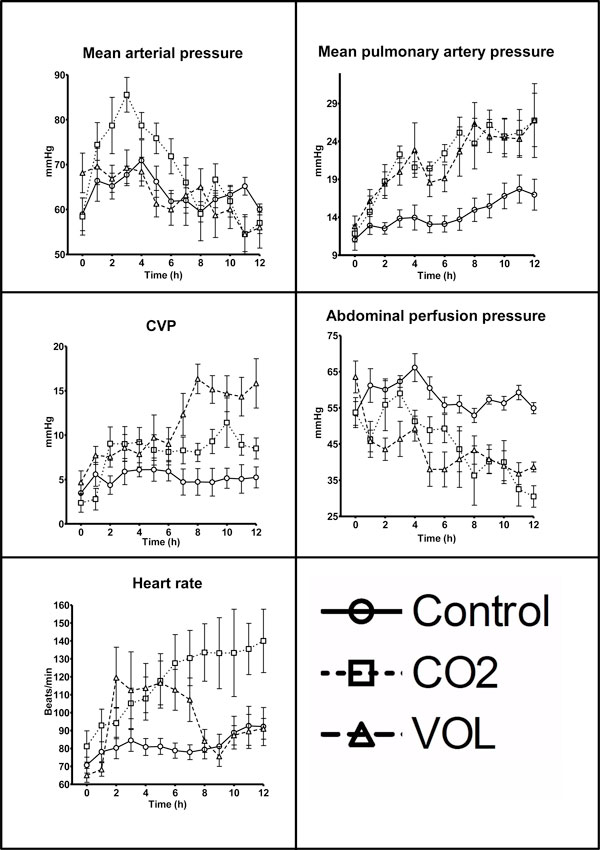
**Cardiovascular parameters**. Data presented as mean ± SEM. Control, control group; CO2, group with CO_2 _pneumoperitoneum; VOL, group with intra-abdominal volume addition by placement of saline bags.

Heart rate was continually increasing in the CO2 group; the VOL group heart rate initially increased and then decreased. The VOL group CVP increased during the last part of the experiment, and MPAP increased in both intervention groups (*P *< 0.0001 for heart rate and CVP, *P *= 0.0195 for MPAP, repeated measures ANOVA. VOL and CO2 group MPAP were different from control at multiple other times than *T *= 12 h (*P *< 0.05, Kruskal-Wallis), data not presented). MAP was increased in the CO2 group during the initial part of the experiment (*P *= 0.0012, repeated measures ANOVA) but later decreased. APP was decreased in both experimental groups (*P *< 0.0001, repeated measures ANOVA) (Table 2, Figure 3).

### Pulmonary function

Respirator settings were changed immediately after IAH induction to maintain or to counteract decreasing tidal volume and saturation and increasing pCO_2_. Cdyn was immediately decreased in both experimental groups and remained stable (*P *< 0.0001, repeated measures ANOVA) (Table 2, Figure 1). At the end of the experiment, the lungs were macroscopically inspected, and the basal posterior parts were darker than the rest of the lungs.

## Discussion

In this study, we investigated whether the choice of a method to create IAH, either CO_2 _pneumoperitoneum or addition of volume to the intra-abdominal cavity, had any impact on the development of organ dysfunction. We observed both the specific organ damage generated and the temporal development of organ damage.

### Main results

Our results showed that both methods of generating IAH produce ACS [5]. Our finding that three animals (38%) died in each group toward the end of the experiment demonstrates that the animals sustained organ damage. The development of organ dysfunction was found to be simultaneous for creatinine increase, K^+ ^increase, MPAP, and Cdyn. Both intervention groups needed an immediate change in respirator settings after IAH generation to ensure sufficient ventilation. Both intervention groups ended up with APP < 50 mmHg, which is below the suggested recommendations [6]. All findings observed changed throughout study, except Cdyn which immediately decreased and then remained stable. The major differences between the models were hypercapnia and acidosis observed in the animals with CO_2 _pneumoperitoneum. Other differences included different temporal development of lactate increase, initial VOL group tachycardia, the VOL group's CVP increase after 8 h, and initially higher MAP in the CO2 group.

Previous studies on IAH, both clinical and experimental, demonstrate an impact on organs similar to our findings. Kidney damage is known to appear as an early sign of organ damage during IAH and ACS [4,17]. Experimentally decreased blood flow in the peritubular capillaries has been shown [10], as well as redistribution of blood away from the kidneys [11] during IAH. Among IAH/ACS patients, impaired respiratory function and the need for mechanical ventilation are well-known complications [3,4,17]. In a previously published study regarding IAH generated by CO_2 _pneumoperitoneum in pigs, all animals survived for 24 h [8], which is a much higher survival rate than we found.

Control animals experienced increased creatinine concentration, heart rate, and CVP. Nevertheless, these were minor changes and probably caused by the sham operative procedures. Accordingly, significant difference in organ damage between the control group and the intervention groups must be caused by IAH. Furthermore, control animals showed no increase in IBP after the sham procedures. Therefore, these animals must have been sedated to such a degree that muscle contractions did not increase the IAP. Any IAH observed in our animals was thus likely to be caused by the CO_2 _insufflations or the placement of extra volume in the experimental groups.

### Comparison of models

The excess pCO_2 _in the CO_2 _group was most likely caused by the absorption of CO_2 _inserted into the abdominal cavity with the laparoscopic insufflator. This effect is already known [14]. All changes in acid-base status occurred despite our attempts to ensure sufficient ventilation, as described in the 'Materials and methods' section. Base excess was lowered almost equally in both our experimental groups. This could mean that the maximum capacity of metabolic compensation for acidosis was reached in the CO2 group. In addition, the anuria precluded metabolic compensatory mechanisms that require functioning kidneys.

After initial IAH generation in the VOL group, we found no further IBP increase. Cheatham et al. stated: 'The more the degree of IAH, the more urgent is the need for decompression of the abdomen...' [6]. Since the animals suffered multiple organ damage, an increase in IBP might be expected to resemble the deteriorating condition. We did not observe this.

Comparing IAP with IBP in the CO2 group showed that IBP was constantly 1 to 2 mmHg above IAP. In the VOL model, the IBP was slightly lower than that in the CO2 group. The reason for this was that the abdominal cavity of our animals could only hold seven saline bags, producing an IBP of 22 mmHg. The lower IBP in the VOL group could result in less impact than if the IBP had been as high as in the CO2 group.

Both methods used to generate IAH are easy to apply. Pneumoperitoneum will rarely be the cause of real-life IAH. The VOL method is therefore probably closer to resembling pathological IAH than the CO_2 _method. Because acidosis is part of MODS, the CO2 model could be used if acidosis is desired in a given experiment. Care must be taken, however, not to confuse artificial with pathologic acidosis.

### Limitations

After approximately 8 h of IAH, unexplained results were observed. Base excess became lower in the CO2 group than in the VOL group; the CO2 group's lactate increased from baseline values; the VOL group heart rate decreased back to baseline level; and CVP increased. We are unable to explain these results, yet we cannot rule out that the model was unstable, which would introduce a bias. Better monitoring of loading conditions in animals by monitoring cardiac output or pulmonary artery occlusion pressure would have provided valuable information that might reveal why heart rate and CVP developed as they did. Measurement of esophageal pressure could provide information regarding the relation between intra-thoracic pressure and the observed changes in CVP. Monitoring cytokines could also have provided valuable information about the animals' condition during ACS development. IBP was not measured as recommended: at the mid-axillary line at the iliac crest with not more than 25 ml of intravesicular volume [5]. We measured IBP at the symphysis pubis and did not have control over the intravesical volume.

We only included eight animals in each group. This small number makes observations vulnerable to bias caused by few outlier animals. Toward the end of the experiment, the animals died in the IAH groups. It may have influenced results that data regarding the sickest animals were not included in the analytical evaluation after their deaths. This could explain the CO2 group's decreasing pCO_2 _and K^+ ^at 12 h.

A drawback of the VOL method is the inability to ensure that the intra-abdominally bags are placed in the same position in every experiment. This may result in nonuniform pressure transmission producing a local pelvic compartment syndrome. Combining IBP and intragastric pressure could provide further information on this issue [18]. Another drawback when using saline bags is that the bags' borders are not smooth. They may cause bleeding or inflammation if the sharp parts interfere with surrounding tissues or vessels. We did not perform post-mortem examinations for organ or vessel damage.

## Conclusion

Our study showed that IAH generated with either of two methods: CO_2 _pneumoperitoneum or intra-abdominal placement of saline bags, causes abdominal compartment syndrome and organ dysfunction within 12 h. The animals subjected to CO_2 _pneumoperitoneum developed artificial hypercapnia and acidosis.

## Abbreviations

ACS: abdominal compartment syndrome; ANOVA: analysis of variance; APP: abdominal perfusion pressure; Cdyn: dynamic lung compliance; CO2: group with IAH with CO_2 _pneumoperitoneum; CVP: central venous pressure; IAH: intra-abdominal hypertension; IAP: intra-abdominal pressure; IBP: intra-bladder pressure; MAP: mean arterial pressure; MODS: multiple organ dysfunction syndrome; MPAP: mean pulmonary artery pressure; pCO_2_: Partial pressure of carbon dioxide;PEEP: positive end-expiratory pressure; *P*_insp_: inspiratory pressure; VOL: group with IAH with intra-abdominal volume addition.

## Competing interests

The authors declare that they have no competing interests.

## Authors' contributions

MM performed the pilot studies, participated in the design of the study and the experiments, performed the experiments, drafted the samples, reviewed and analyzed the data, performed some of the statistical analysis, and wrote and submitted the manuscript. UKK took part in designing the experiments, in performing the pilot studies and experiments, and in drafting the samples. JRL participated in the design of the study and the experiments, designed the anesthesiological setup for the experiments, performed some of the statistical analysis and graphical presentations, and critically revised the manuscript. HBS and LBS took part in the design of the study and the experiments and critically revised the manuscript. All authors' contributions meet the criteria to justify authorship. All authors read and approved the final manuscript.

## Endnotes

The work was performed at the Institute of Clinical Medicine, Aarhus University, Aarhus University Hospital, Skejby, Denmark.
